# Microbiome, Epigenetics, and Nutritional Factors in Shaping Perinatal Pregnancy Outcomes

**DOI:** 10.3390/ijms27041622

**Published:** 2026-02-07

**Authors:** Miljana Z. Jovandaric, Sandra Babic, Milos Milincic, Biljana Medjo, Misela Raus, Mirjana Krstic, Dejan Tiric

**Affiliations:** 1 Department of Neonatology, Clinic for Gynecology and Obstetrics, University Clinical Center of Serbia, 11000 Belgrade, Serbia; 2 Department of Obstetrics and Gynecology, Clinic for Gynecology and Obstetrics, University Clinical Center of Serbia, 11000 Belgrade, Serbiamilosmilincic@gmail.com (M.M.); 3 Pediatric and Neonatal Intensive Care Unit, University Children’s Hospital, 11000 Belgrade, Serbia; 4 Faculty of Medicine, University of Belgrade, 11000 Belgrade, Serbia; 5 Department of Neonatology, University Children’s Hospital, 11000 Belgrade, Serbia; 6 Hospital for Gynecology and Obstetrics, Clinical Hospital Center Zemun, 11080 Zemun, Serbia; 7 Department of Obstetrics and Gynecology, University Clinical Hospital Mostar, 88000 Mostar, Bosnia and Herzegovina; 8 Faculty of Medicine, University of Mostar, 88000 Mostar, Bosnia and Herzegovina

**Keywords:** microbiome, nutrition, epigenetic, oxidative stress, perinatal outcome

## Abstract

Maternal nutrition and gut microbiome composition are central regulators of fetal development and perinatal outcomes, modulating immune signaling, oxidative balance, and epigenetic programming. The authors searched PubMed, Scopus, the Cochrane Library, and Web of Science for full-text, peer-reviewed articles published in English between 2010 and 2025, using keywords relevant to maternal diet, gut microbiome, epigenetic modifications, oxidative stress, reactive oxygen species (ROS), short-chain fatty acids (SCFAs), placental function, and perinatal outcomes. Selected studies provided detailed insights into how maternal dietary patterns and microbiome-derived metabolites influence placental function, fetal growth, and neonatal health. Integration of the microbiome, epigenetics, and nutritional factors reveals key molecular and metabolic networks that shape perinatal health. Targeted modulation of these networks provides a foundation for personalized strategies to improve neonatal outcomes.

## 1. Introduction

The perinatal period is a vital stage of fetal and early neonatal development, during which complex immunometabolic, epigenetic, and biochemical mechanisms are established, directly affecting growth, tissue differentiation, and adaptation to both intrauterine and extrauterine environments [1]. This period includes the late second and third trimesters of pregnancy, labor, and the initial weeks after birth. It is characterized by rapid organ formation, vascular remodeling, and functional development of the liver, lungs, heart, and brain, along with the growth of immune and endocrine systems [2]. Biochemical processes are closely regulated to ensure optimal lipid, glucose, amino acid, and antioxidant metabolism, supporting energy balance, macromolecule synthesis, and protection against oxidative stress [3]. Key signaling pathways—such as nuclear factor kappa-light-chain-enhancer of activated B cells (NF-κB), mitogen-activated protein kinase (MAPK), nuclear factor erythroid 2-related factor 2 (Nrf2), adenosine monophosphate-activated protein kinase (AMPK), peroxisome proliferator-activated receptor alpha/gamma (PPAR-α/γ), sterol regulatory element-binding protein-1c (SREBP-1c), liver X receptor (LXR), and peroxisome proliferator-activated receptor gamma coactivator 1-alpha (PGC-1α)—regulate the expression of pro- and anti-inflammatory cytokines, antioxidant enzymes, and lipid metabolism factors. Simultaneously, epigenetic mechanisms—including DNA methylation, histone acetylation, and non-coding RNAs such as microRNAs (miRNA) and long non-coding RNAs (lncRNA)—provide precise control over immunometabolic activity, oxidative stress, and lipid balance [4,5]. Collectively, these processes establish the foundation for postnatal adaptation, long-term health, and metabolic flexibility of the newborn [6].

Maternal microbiota exerts a profound influence on the intrauterine immunometabolic environment and fetal development [7]. The vaginal microbiome, predominantly composed of Lactobacillus species, maintains an acidic environment and modulates NF-κB and MAPK signaling in the endometrium and fetal membranes. Dysbiosis of the vaginal or gastrointestinal microbiome can trigger pro-inflammatory expression of cyclooxygenase-2 (COX-2), inducible nitric oxide synthase (iNOS), and cytokines, including tumor necrosis factor-alpha (TNF-α), interleukin-6 (IL-6), and interleukin-1 beta (IL-1β), directly affecting the inflammatory milieu and fetal lipid metabolism [8,9]. The placental microbiome further regulates cytokine expression and the activation of antioxidant enzymes, including superoxide dismutase 1 and 2 (SOD1, SOD2), glutathione peroxidase 1 and 4 (GPx1, GPx4), catalase, and heme oxygenase-1 (HO-1). Additionally, the oral microbiome modulates lipid metabolism through Toll-like receptor 4 (TLR4) signaling and endotoxin stimulation, affecting the expression of PPAR-α, PPAR-γ, SREBP-1c, and LXR [10,11]. The gastrointestinal microbiome generates short-chain fatty acids (SCFAs)—acetate, propionate, and butyrate—that serve simultaneously as metabolic substrates, signaling molecules, and epigenetic modulators, integrating immunometabolic, antioxidant, and epigenetic networks [12,13].

Among SCFAs, butyrate inhibits histone deacetylases (HDACs), enhancing acetylation of histones H3 and H4 at promoter regions of genes encoding anti-inflammatory cytokines and antioxidant enzymes [14]. Acetate, converted into acetyl-coenzyme A (acetyl-CoA), serves as a substrate for lipogenesis, cholesterol synthesis, histone acetylation, and protein acetylation. In contrast, propionate enters the Krebs cycle via succinyl-CoA, promoting gluconeogenesis, adenosine triphosphate (ATP) production, and mitochondrial energy generation. SCFAs also activate G protein-coupled receptors 41 and 43 (GPR41, GPR43), stimulate AMPK and PGC-1α signaling, enhance fatty acid β-oxidation and mitochondrial biogenesis, increase antioxidant enzyme expression, and stabilize the mitochondrial membrane, while simultaneously modulating epigenetic processes [15].

Epigenetic regulation is essential during the perinatal period and includes DNA methylation of CpG islands in promoter regions of immunometabolic and lipid metabolism genes, mediated by DNA methyltransferases 1, 3A, and 3B (DNMT1, DNMT3A, DNMT3B), a process strongly influenced by maternal nutritional status, particularly folate and vitamin B12 intake [16]. Histone modifications control accessibility for transcription factors such as NF-κB, Nrf2, and PPAR-α/γ, fine-tuning inflammatory responses and lipid metabolism. MicroRNAs (miRNA-146a, miRNA-155, miRNA-21, miRNA-33a/b, miRNA-122) and lncRNAs (H19, MEG3, MALAT1, NEAT1) tightly regulate cytokine expression, lipid enzymes, and antioxidant proteins [17,18]. These epigenetic modifications are particularly sensitive during the perinatal period, as they become permanently inscribed in the epigenetic imprint, shaping the newborn’s metabolism, immune profile, and lipid homeostasis [19].

Fetal lipid metabolism is orchestrated by the interplay of PPAR-α/γ, SREBP-1c, LXR, and AMPK signaling pathways [20]. PPAR-α promotes fatty acid β-oxidation, PPAR-γ regulates lipoprotein lipase and triglyceride synthesis, SREBP-1c drives lipogenesis, and LXR regulates cholesterol transport and high-density lipoprotein (HDL) particle formation. AMPK inhibits acetyl-CoA carboxylase (ACC) and fatty acid synthase (FAS) while activating carnitine palmitoyltransferase 1 (CPT1) and fatty acid oxidation, preserving energy balance. SCFAs and omega-3 fatty acids, eicosapentaenoic acid (EPA) and docosahexaenoic acid (DHA), modulate these pathways, suppress NF-κB and MAPK-mediated pro-inflammatory cascades, and activate Nrf2-dependent antioxidant mechanisms. This integrated network couples lipid metabolism, inflammation, and oxidative stress into a coherent immunometabolic system. Understanding these mechanisms provides a foundation for innovative perinatal interventions, including personalized nutritional and microbiome-based strategies, aiming to reduce complications and improve neonatal health outcomes [2].

## 2. Literature Search Strategy and Data Sources

A literature search was performed to assess the impact of maternal nutrition and gut microbiome composition on fetal development and perinatal outcomes. PubMed, Scopus, Cochrane Library, and Web of Science were searched for peer-reviewed articles in English published between 2010 and 2025 using keywords including “maternal diet”, “gut microbiome”, “epigenetic modifications”, “oxidative stress”, “reactive oxygen species”, “short-chain fatty acids”, “placental function”, and “perinatal outcomes”.

Studies addressing maternal dietary patterns, microbiome-derived metabolites, or epigenetic and oxidative-inflammatory mechanisms that affect placental function, fetal growth, and neonatal outcomes were included. Non-English articles, articles with unavailable full texts, or those lacking translational relevance were excluded. Selected manuscripts were organized into Section 3, focusing on maternal and neonatal microbiome; Section 4, detailing molecular mechanisms including signaling pathways, epigenetic regulation, and fetal protection from oxidative stress; Section 5, discussing nutritional factors and their molecular interactions

## 3. Maternal and Neonatal Microbiome in Perinatal Outcomes

### 3.1. Maternal Microbiome Dynamics During Pregnancy

Pregnancy represents a period of profound physiological transformation, during which the maternal microbiome assumes a central regulatory role, influencing both maternal and fetal health [21]. The dynamic interplay between oral, gut, and vaginal microbial communities, maternal diet, hormonal fluctuations, and immune adaptation orchestrates metabolic, inflammatory, oxidative, and epigenetic processes that collectively shape perinatal outcomes.

The oral cavity harbors complex microbial communities, including Streptococcus, Fusobacterium, and Veillonella, functioning both as a reservoir of commensal microbes and a source of pathogen-associated molecular patterns (PAMPs) such as lipopolysaccharide (LPS) and peptidoglycans [22]. These microbial components can translocate systemically, engaging Toll-like receptors 2 and 4 (TLR2, TLR4) on trophoblasts and maternal immune cells. Activation of NF-κB and MAPK pathways orchestrates a finely tuned cytokine environment, balancing pro-inflammatory mediators (TNF-α, IL-6, IL-1β) with regulatory cytokines such as IL-10 [23]. When balanced, this inflammatory milieu supports vascular remodeling, uterine adaptation, trophoblast invasion, and optimal nutrient delivery. Persistent low-grade inflammation, often exacerbated by periodontal infections or oral dysbiosis, disrupts placental lipid metabolism, enhances COX-2 expression, increases prostaglandin E2 synthesis, and elevates preterm birth risk [24]. Oral dysbiosis has also been associated with systemic lipid alterations, including elevated triglycerides and cholesterol, further influencing placental nutrient handling [25].

The gut microbiome undergoes dynamic remodeling throughout gestation. Early pregnancy is characterized by increases in Bacteroidetes and Firmicutes populations, capable of producing SCFAs. such as butyrate, acetate, and propionate [26]. SCFAs act as critical signaling molecules interfacing with maternal immune and metabolic networks. Butyrate activates G-protein-coupled receptors GPR41 and GPR43, inhibits HDACs, promotes Treg differentiation, upregulates anti-inflammatory cytokines IL-10, TGF-β, and suppresses NF-κB transcription [27]. It also engages Nrf2, inducing antioxidant enzymes SOD and GPx to mitigate placental oxidative stress [28]. Propionate and acetate modulate MAPK and ERK signaling, influencing placental vascular tone, nutrient transport, fetal lipid homeostasis, and gluconeogenesis [29]. Dysbiosis, triggered by maternal stress, antibiotics, infection, or poor nutrition, reduces SCFA production, enhances Th17 pro-inflammatory responses, increases oxidative stress, and destabilizes the maternal-fetal interface, potentially compromising fetal growth and predisposing to gestational diabetes and preeclampsia [30,31,32,33,34,35].

The vaginal microbiome, predominantly Lactobacillus species such as *L. crispatus* and *L. gasseri*, maintains low pH and produces bacteriocins and H_2_O_2_, preventing pathogen colonization [36]. Sex hormones regulate glycogen deposition in vaginal epithelium, supporting lactobacilli growth [37]. Disruption via bacterial vaginosis, sexually transmitted infections, or antibiotics elevates IL-1β and IL-8, increases epithelial permeability, activates local MAPK signaling, and raises susceptibility to ascending infections and chorioamnionitis [38]. Vaginal dysbiosis also alters prostaglandin and cytokine profiles in amniotic fluid, impacting placental and fetal physiology [39].

Maternal diet is a key determinant of microbiome composition and function. Fiber-rich, omega-3–rich, and micronutrient-dense diets support microbial diversity, SCFA production, anti-inflammatory signaling, and antioxidant defenses [40]. Conversely, high saturated fat, simple sugars, and processed foods promote pathobiont overgrowth, endotoxemia, and NF-κB–mediated inflammation, disrupting prostaglandin signaling, placental lipid metabolism, and nutrient transport [41]. Adequate micronutrient status synergizes with progesterone-mediated immunotolerance, supporting placental vascular remodeling, Treg differentiation, antioxidant enzyme function, and DNA methylation [32].

Progesterone and estrogen fluctuations throughout pregnancy modulate gut motility, bile acid secretion, mucosal immunity, and microbial activity, collectively affecting SCFA availability [42]. Increased intestinal permeability in late pregnancy allows controlled translocation of SCFAs into systemic circulation, facilitating maternal gut–liver–placenta cross-talk [43]. SCFAs regulate endothelial cells, hepatocytes, immune populations, and placental trophoblasts via HDAC inhibition and GPCR engagement. Butyrate modulates lipid synthesis through AMPK and PPAR-γ, optimizing fetal nutrient supply, while propionate and acetate influence gluconeogenesis, lipid oxidation, and prostaglandin synthesis via COX-2 expression [44].

Maternal systemic immune adaptation is closely tied to microbial dynamics. TLR-mediated detection of microbial components triggers a balanced cytokine response essential for fetal tolerance. Excessive NF-κB or MAPK activation increases TNF-α and IL-6, destabilizes lipid metabolism, elevates oxidative stress, and increases prostaglandin E2, affecting uterine contractility [45]. SCFAs counter pro-inflammatory signals, enhancing Nrf2-mediated transcription of antioxidant enzymes, reducing ROS accumulation, and supporting placental endothelial function [46]. Epigenetically, SCFAs act as HDAC inhibitors, increasing histone acetylation at promoters of anti-inflammatory genes (Foxp3, IL-10), reinforcing Treg differentiation and maternal-fetal immune tolerance [47,48]. DNA methylation patterns, influenced by microbial metabolites and micronutrients, affect cytokine production, prostaglandin synthesis, oxidative stress, and lipid metabolism [35,49].

Placental oxidative homeostasis is regulated by maternal microbiota, SCFAs, and antioxidant pathways. Nrf2 activation increases GPx, SOD, and catalase expression, mitigating ROS. Dysbiosis elevates ROS, activates NF-κB, boosts COX-2, and disrupts trophoblast lipid metabolism, affecting prostaglandin profiles, uterine contractions, fetal growth, and lipid balance [50]. SCFAs reach the liver via the portal vein, modulating lipid synthesis, gluconeogenesis, and bile acid metabolism, thereby impacting placental nutrient transfer. Butyrate and propionate influence hepatic AMPK and PPAR-γ signaling, ensuring balanced essential fatty acid provision to the fetus [49,51,52,53].

### 3.2. Vertical Microbiome Transmission and Maternal–Fetal Interactions

The establishment of the neonatal microbiome begins in utero, profoundly influenced by maternal microbial communities and their metabolic signaling [54]. While the historical concept of a sterile womb suggested fetal exposure to microbes only after birth, current evidence demonstrates that microbial DNA, metabolites, and extracellular vesicles traverse the maternal–fetal interface, shaping fetal immune and metabolic ontogeny [55,56]. Vaginal, gut, and oral microbiota contribute to this vertical transmission, delivering not only live microbes but also microbial metabolites, including SCFAs, LPS, peptidoglycans, and extracellular vesicles, which modulate fetal immune development through placental signaling [57,58]. The placenta functions as a dynamic immunometabolic organ, expressing pattern-recognition receptors such as TLRs and NOD-like receptors that sense microbial components and translate these signals into cytokine and prostaglandin outputs, thereby influencing fetal immune programming, vascular remodeling, and metabolic adaptation [59,60].

During the process of vaginal delivery, neonates encounter an intricate maternal microbiota, predominantly comprising Lactobacillus, Bifidobacterium, and Prevotella, which orchestrates the initial establishment of the infant’s microbiome and constitutes a critical determinant for the maturation of the neonatal immune system [61]. This initial colonization is instrumental in educating the innate immune components, engaging pattern-recognition receptors that activate dendritic cells, macrophages, and innate lymphoid populations, thereby establishing an early framework for immunological homeostasis [62]. Maternal SCFAs—notably butyrate, propionate, and acetate—traverse the placental barrier and modulate fetal Nrf2 signaling pathways, resulting in upregulated expression of antioxidant enzymes and attenuation of NF-κB–driven pro-inflammatory responses [63]. Furthermore, butyrate and propionate function as histone deacetylase inhibitors at key loci such as Foxp3 and IL-10, epigenetically facilitating the differentiation of regulatory T cells and reinforcing long-term immunotolerance [64]. Perturbations in vertical microbial transmission, whether through cesarean section, maternal dysbiosis, or intrapartum antibiotic administration, can markedly alter microbial seeding, diminish SCFA availability, and skew neonatal immune responses toward Th1/Th17 phenotypes, thus predisposing to a heightened risk of inflammatory, allergic, or metabolic disorders throughout postnatal life [65].

Beyond their immunomodulatory effects, maternal SCFAs have a profound influence on placental lipid metabolism and prostaglandin synthesis, which are essential for optimal fetal growth trajectories and the maintenance of uteroplacental vascular function [66]. Specifically, butyrate and propionate regulate AMPK and PPAR-γ signaling in trophoblast cells, ensuring a delicate equilibrium between triglyceride storage, fatty acid oxidation, and cholesterol transport [32]. Simultaneously, microbiota-derived metabolites modulate COX-2 expression and prostaglandin production, thereby impacting uteroplacental perfusion, nutrient delivery, and fetal metabolic programming [67]. Disruptions to these finely tuned pathways, induced by maternal stress, dysbiosis, or inadequate nutritional status, result in aberrant activation of NF-κB and MAPK cascades, excessive secretion of pro-inflammatory cytokines such as TNF-α and IL-6, and altered prostaglandin profiles, collectively compromising fetal lipid handling, redox homeostasis, and immunological tolerance [68,69].

This integrative crosstalk between maternal microbiota, SCFA-mediated signaling, and placental metabolic processes underscores the mechanistic basis for observed correlations between maternal microbial composition and neonatal outcomes, highlighting potential translational avenues for targeted interventions aimed at optimizing perinatal health and enhancing resilience to postnatal immune and metabolic challenges [70].

The maternal microbiome, particularly in oral and gastrointestinal niches, can contribute to vertical transmission of microorganisms and their metabolic products via systemic circulation and potential interaction with the fetoplacental interface. Studies indicate that maternal microbiome composition can influence fetal gene expression involved in immune response, energy metabolism, and neurophysiological pathways already during the intrauterine period [71].

Maternal conditions during pregnancy shape the microbiome composition and can influence vertical microbial transmission and fetoplacental interactions. Obesity is associated with increased abundance of Bacteroides and Staphylococcus, while maternal dysbiosis modulates SCFA production, metabolism, and host gene expression, indirectly affecting infant development and microbiome establishment. These changes may increase the risk of metabolic disorders in the offspring [72]. Microbiome changes throughout life, with neonates exhibiting a simple but plastic microbiota dominated by Bifidobacterium, while diversity increases in early childhood due to the introduction of solid foods and environmental exposure. In adulthood, the microbiome is relatively stable and individual-specific, yet remains influenced by diet, stress, and medications. In older adults, diversity decreases, protective species are lost, and opportunistic bacteria dominate, contributing to inflammation and metabolic dysregulation. These changes reflect a complex interplay between the microbiome, immunity, and host metabolism across the lifespan [73]. Polycystic ovary syndrome (PCOS) is associated with changes in the gut and genital microbiome, which contribute to metabolic dysfunction, inflammation, and hormonal imbalance. Women with PCOS often experience reduced microbiota diversity and a reduction in protective strains. At the same time, dysbiosis affects hormone metabolism, inflammatory pathways, and SCFA production, which may contribute to the development of insulin resistance, obesity, and reproductive complications. The changes include increased abundance of Bacteroides, Prevotella, Proteobacteria, Firmicutes, and Enterobacteriaceae and may shape the early development of infants and the establishment of their microbiome [74]. Endometriosis is associated with disturbances in the gut and genital microbiome, contributing to inflammation and immune dysregulation. Gut dysbiosis increases epithelial barrier permeability, facilitating bacterial translocation and promoting the persistence and progression of endometrial implants. Changes in microbiome composition, including Bacteroides, Prevotella, Proteobacteria, and Enterobacteriaceae, reflect a bidirectional interaction between the microbiome and the disease [75].

Experimental studies have shown that elimination of the maternal gut microbiome in mice results in reduced placental growth, suggesting that the microbiome contributes to placental development through alterations in vascularization and structure. Although direct human evidence remains limited, an increasing number of reviews suggest that maternal microbiome dysbiosis—including oral, vaginal, and gut components—may be associated with pregnancy complications such as gestational diabetes, preeclampsia, and preterm birth [76].

The vaginal microbiota maintains an acidic environment through the production of lactic acid and hydrogen peroxide, preventing colonization by pathogenic microorganisms and modulating local cytokine responses. Alterations in the vaginal niche—such as decreased *Lactobacillus* spp. dominance—can lead to increased proinflammatory expression and oxidative stress, which may be transmitted via the fetoplacental interface to the fetus. The concept of the “gut–placenta axis” is gaining increasing attention, highlighting the interconnection of gut and vaginal microbiota with intrauterine development [77].

Maternal nutritional status acts synergistically with microbial activity to fine-tune fetal epigenetic programs. A diet rich in dietary fibers stimulates the production of SCFA, which modulate histone acetylation and support anti-inflammatory gene expression [78]. Methyl-donor micronutrients—folate, vitamin B12, and choline—play a central role in DNA methylation and modulation of gene expression in fetal immune and metabolic tissues [79]. Micronutrients such as selenium, zinc, and vitamin D support antioxidant enzymes and regulatory T cell (Treg) differentiation, potentially reducing maternal systemic inflammation and improving the intrauterine environment for the fetus [80]. Meta-analyses confirm that nutritional deficiencies and microbiome dysbiosis can enhance NF-κB and MAPK pathway activation, destabilize lipid metabolism, and increase the neonate’s predisposition to metabolic syndrome and immune dysregulation [33]. Overall, the integration of microbial, nutritional, and epigenetic mechanisms provides a robust translational framework for understanding perinatal outcomes and underscores the influence of the maternal gut microbiome on the fetal gut–brain axis and long-term health [81].

Placental oxidative balance represents a pivotal determinant of fetoplacental health, intricately governed by the maternal microbiota, microbial metabolites, particularly SCFAs, and the dynamic interplay of endogenous antioxidant pathways. Perturbations in the maternal gut ecosystem during gestation provoke systemic inflammatory cascades and excessive generation of reactive oxygen species (ROS), culminating in trophoblastic dysfunction and altered placental perfusion. Such redox disequilibrium is tightly linked to obesity, gestational diabetes, and hypertensive disorders of pregnancy, wherein the placenta exhibits mitochondrial distress and impaired detoxifying capacity [82].

Among the key regulators, SCFAs—especially butyrate and propionate—activate the Nrf2 signaling pathway, which orchestrates the transcription of genes encoding antioxidant and phase II detoxification enzymes. When maternal dysbiosis limits SCFA bioavailability, Nrf2-dependent cytoprotection is blunted, leading to unchecked oxidative injury in trophoblasts and endothelial cells, thereby compromising nutrient and oxygen exchange between mother and fetus [83].

This pro-oxidative milieu further induces lipid peroxidation and the release of bioactive aldehydes, which amplify inflammatory signaling through activation of NF-κB and MAPK pathways, perpetuating cytokine production and leukocyte infiltration into placental tissue. Such sustained inflammatory feedback loops represent a critical mechanistic bridge between microbiota imbalance and adverse perinatal outcomes, including preterm birth and intrauterine growth restriction [49].

Effective antioxidative defense depends on coordinated upregulation of GPx, SOD, and catalase, enzymes whose expression and activity are epigenetically regulated in response to redox-sensitive transcription factors. Disturbances in this enzymatic triad result in accumulation of hydrogen peroxide and peroxynitrite, thereby exacerbating oxidative stress–mediated endothelial damage and placental senescence [84].

Importantly, SCFAs not only act as metabolic substrates but also as epigenetic modulators through inhibition of HDACs, promoting chromatin relaxation and transcription of genes critical for redox homeostasis, immune tolerance, and metabolic programming of the fetus. These molecular mechanisms underscore the central role of the maternal microbiota–metabolite axis in maintaining placental integrity and protecting against oxidative and inflammatory insults throughout gestation [51,85].

The integration of vertical microbiome transmission, SCFA-mediated signaling, epigenetic modulation, prostaglandin regulation, lipid metabolism, and oxidative stress forms a complex, multilayered network shaping neonatal immune and metabolic outcomes. Dysregulation at any node—through dysbiosis, maternal malnutrition, or stress—can propagate systemic inflammation, impair nutrient delivery, and compromise fetal epigenetic programming. Conversely, optimal maternal microbiome composition, adequate SCFA production, and sufficient micronutrient intake maintain a protective, anti-inflammatory intrauterine environment, establishing a foundation for robust neonatal immunity and metabolic resilience [79,86].

### 3.3. Maternal Microbiome Dysbiosis, Hormonal Interactions, and Translational Implications in Pregnancy

Maternal microbiome dysbiosis represents a fundamental pathophysiological modulator that disrupts immunological, metabolic, and epigenetic homeostasis within the gestational environment, wherein alterations in microbial composition, characterized by a reduction in beneficial genera such as Lactobacillus and Bifidobacterium, alongside an increase in opportunistic bacteria, activate complex immunometabolic and hormonal signaling networks [87].

During pregnancy, remodeling of the maternal microbiome promotes alterations in cytokine networks at the maternal–fetal interface, including changes in TNF-α, IL-6, and IL-10, which in turn favor differentiation of immune cells toward Treg and Th2 phenotypes, thereby establishing a tolerogenic immunological milieu [88].

Dysbiosis may stimulate TLR-4 expressed on trophoblasts and decidual immune cells, triggering activation of NF-κB and MAPK (p38) signaling pathways and initiating pro-inflammatory transcriptional programs [89].

Activation of NF-κB signaling enhances the expression of pro-inflammatory cytokines and may upregulate matrix metalloproteinase-9 (MMP-9) as well as COX-2, thereby amplifying placental inflammation, impairing vascular remodeling, and potentially contributing to pregnancy complications such as preterm birth [90].

Simultaneously, the NRF2 transcription factor functions as a critical protective component of oxidative homeostasis by inducing the expression of antioxidant enzymes (SOD, catalase, glutathione peroxidase) and safeguarding placental cells from oxidative damage. Dysbiosis may compromise this defense, associated with oxidative stress and placental dysfunction, particularly in the context of maternal metabolic disorders [91]. SCFAs produced by the gut microbiota play a central role in this system: including butyrate, propionate, and acetate modulate autophagy, signaling, and epigenetic regulation in the placenta, and their circulating levels in the mother correlate with maternal metabolic parameters and offspring anthropometric characteristics [92].

Experimental models indicate that butyrate supplementation during pregnancy can protect offspring from intestinal inflammatory injury, suggesting long-term health benefits [93].

During pregnancy, SCFAs derived from the maternal microbiota exert epigenetic effects through inhibition of HDACs, leading to increased histone acetylation at regulatory loci such as the FOXP3 promoter, thereby enhancing regulatory Treg. differentiation and stabilizing a tolerogenic immune profile [94].

Furthermore, pregnancy hormones—particularly progesterone and estrogen—dynamically interact with the maternal microbiome, modulating its composition and metabolic output. In turn, microbiota-derived SCFAs act on placental and maternal tissues via G-protein-coupled receptors (e.g., GPR43, GPR41) and epigenetic mechanisms, influencing enzymatic pathways in the placenta, including steroidogenesis and cortisol synthesis, thereby establishing a bidirectional regulatory circuit between sex hormones and microbial metabolites [44].

From a translational perspective, dysbiosis of the maternal microbiome during gestation, especially when coupled with hormonal imbalance, may increase the risk of adverse outcomes, such as preterm birth, intrauterine growth restriction, placental insufficiency, and later immunometabolic dysregulation in the offspring [32]. A deeper mechanistic understanding of these interactions paves the way for interventions, such as probiotic or postbiotic supplementation, dietary fiber enhancement, or hormonal modulation, aimed at restoring microbiome hormonal homeostasis and improving both maternal and neonatal health outcomes [95].

#### Maternal Milk and Neonatal Microbiome Development

Human breast milk is increasingly recognized not merely as a source of nutrition but as a dynamic biological system reflecting maternal microbiome composition, immune status, and nutritional environment, thereby shaping neonatal microbial colonization, immune development, and long-term health outcomes [28,96]. Its microbial content, once considered contamination, represents a deliberate inoculum including Lactobacillus, Bifidobacterium, Streptococcus, and Staphylococcus, transferred via the entero-mammary pathway and infant suckling. These microbes establish early symbiotic communities in the neonatal gut, operating in concert with host-derived immunoglobulins, antimicrobial peptides, and immune factors such as secretory IgA, lactoferrin, lysozyme, defensins, cytokines, chemokines, and extracellular vesicles carrying functional microRNAs. Together, these elements create a finely regulated ecological niche that fosters beneficial microbial populations while restraining pathogens [97,98,99,100,101]. IgA limits pathogen adhesion, lactoferrin sequesters iron, and cytokines, including TGF-β, IL-10, and IL-7, promote regulatory T cell expansion and thymic development, supporting a balanced neonatal immune environment [102,103]. These factors ensure that neonatal immunity remains balanced, reducing early predisposition to allergies, auto-immunity and chronic inflammatory disorders [104].

Human milk oligosaccharides (HMOs), the third most abundant component of milk solids, function as selective prebiotics for Bifidobacterium infantis and related taxa, stimulating SCFA. production, including acetate, propionate, and butyrate. These metabolites reinforce intestinal barrier integrity, regulate oxidative stress, and provide substrates for epigenetic programming, with butyrate promoting histone acetylation in genes involved in immune tolerance and propionate and acetate modulating lipid metabolism and energy balance via G-protein-coupled receptors. HMOs additionally act as soluble decoys preventing pathogen adhesion, while maternal secretor status drives inter-individual differences in HMO composition and subsequent neonatal microbial trajectories [105,106,107,108,109].

Lipids constitute nearly half of the caloric content of breast milk and contribute beyond energy provision. Long-chain polyunsaturated fatty acids (LC-PUFAs), including DHA and arachidonic acid, integrate into neural and retinal membranes, while milk fat globule membranes deliver sphingolipids and glycoproteins with immunomodulatory functions [110,111]. Breast milk also provides antioxidant defense via vitamins A, C, and E, glutathione, and enzymatic systems such as catalase and superoxide dismutase, protecting the neonate from oxidative stress during rapid growth [112]. Maternal dysbiosis can reduce SCFA availability, alter lipid quality, and compromise antioxidant capacity, predisposing neonates to metabolic imbalance [113].

Maternal nutrition further modulates breast milk composition and bioactivity. Diets rich in fiber, plant polyphenols, and omega-3 fatty acids enhance SCFA production and transfer, supporting neonatal regulatory immune networks and cognitive development. Conversely, maternal obesity, high-fat diets, or chronic stress disrupt microbial balance, alter HMO composition, and increase proinflammatory cytokines, heightening neonatal risks for necrotizing enterocolitis, atopic sensitization, and later-life metabolic disorders [28,88,114,115].

**Figure 1:**
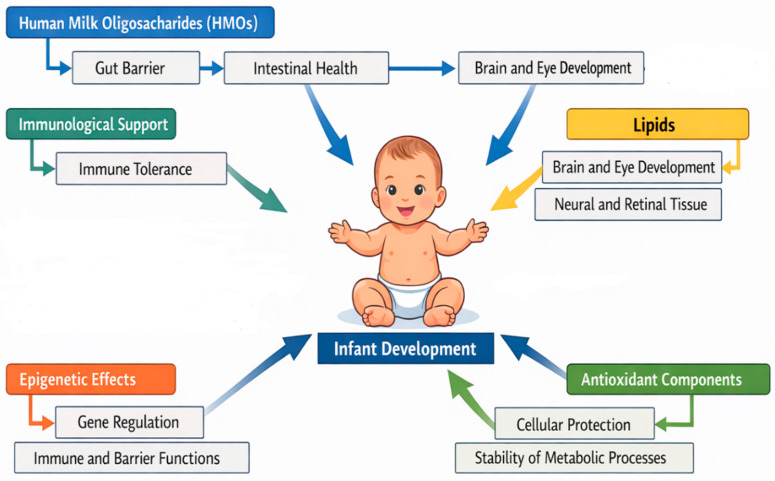
Human Milk Components in Neonatal Development.

Beyond nutrients and microbes, breast milk carries epigenetic information. MicroRNAs, methyl donors such as folate, choline, and vitamin B12, and SCFAs act as epigenetic modulators, influencing neonatal gene expression, lipid metabolism, intestinal growth, and cytokine production. This integration of microbial, immune, metabolic, and epigenetic signals enables breast milk to function as a living interface, bridging maternal microbiome and nutrition with neonatal developmental trajectories [103,116,117,118,119] (Figure 1).

Human milk oligosaccharides (HMOs) shape the composition and functional activity of the neonatal gut microbiome, maintaining intestinal barrier integrity, and gut-derived signaling directly influences neonatal neurodevelopment. Milk lipids contribute to the development of neuronal and retinal tissues. Immunological bioactive factors, including immunoglobulins and cytokines, play a crucial role in supporting the maturation and function of the neonatal immune system. Epigenetic effects of human milk regulate gene expression via DNA methylation, histone modifications, and non-coding RNAs, thereby modulating postnatal development at the molecular level. Antioxidant components reduce oxidative stress and protect cells, providing adequate cellular protection during the critical neonatal period.

## 4. Molecular Mechanisms: Signaling Pathways, Epigenetic Regulation, and Fetal Protection from Oxidative Stress

During pregnancy, the maternal microbiome modulates signaling pathways that maintain immune balance and control oxidative stress in fetal cells. Activation of NF-κB and MAPK induces the release of cytokines, including TNF-α and IL-6, which increases the production of ROS [120]. Elevated ROS causes damage to the membranes, proteins, and mitochondria of the fetus. The anti-inflammatory cytokine IL-10 inhibits NF-κB and MAPK signaling, reducing ROS accumulation and preventing oxidative damage [121].

Nrf2 is released from the cytoplasmic complex with Keap1 and translocates through the NPC into the nucleus, where it binds to the ARE and activates transcription of SOD, GPx, and CAT. These enzymes neutralize ROS and protect DNA, mitochondria, and fetal membranes [122].

Microbiome-derived metabolites further modulate the antioxidant landscape and epigenetic regulation. SCFAs, including butyrate and propionate, inhibit HDACs, thereby opening chromatin and enhancing the expression of antioxidant and anti-inflammatory genes. SCFA also stimulates Nrf2 and reduces NF-κB and MAPK activity, providing both signaling and epigenetic control of oxidative stress [123]. Additionally, tryptophan metabolites and secondary bile acids activate AhR and PPAR-γ, inducing the expression of detoxifying enzymes and protecting membranes from lipid peroxidation [124].

SCFAs play a critical role in the epigenetic regulation of immune cells during implantation. Particularly, butyrate acts as an HDAC inhibitor, modifying chromatin structure and thereby influencing the expression of genes involved in immune regulation. These epigenetic modifications lead to increased expression of anti-inflammatory cytokines, including IL-10 and TGF-β, and induce the activity of IDO. IDO catabolizes tryptophan and inhibits T lymphocyte proliferation, creating a tolerogenic microenvironment for the embryo and supporting successful implantation and early pregnancy development [80].

The maternal reproductive tract microbiome plays a crucial role in modulating immune tolerance during embryo implantation. A stable endometrial and vaginal microbiome, particularly dominated by Lactobacillus species, is associated with improved outcomes in in vitro fertilization (IVF) and frozen embryo transfer (FET), likely through maintenance of an anti-inflammatory microenvironment and regulation of local immune cell populations [125]. Dysbiosis, characterized by reduced Lactobacillus dominance and increased abundance of anaerobic or pathogenic bacteria, correlates with increased implantation failure and early pregnancy loss, potentially due to altered interactions among antigen-presenting cells, natural killer cells, and regulatory T cells (Treg). However, the precise mechanistic pathways remain incompletely understood [126]. These findings highlight the complex interplay between reproductive tract microbiota and maternal immune regulation, suggesting that modulation of microbial communities could represent a target for improving reproductive outcomes, though clinical translation remains in an early stage [127].

### Epigenetic Mechanisms in Perinatal Programming

Histones are protein components of chromatin that enable the organization of DNA into structures known as nucleosomes; this organization regulates gene accessibility for transcription, replication, and DNA repair. Post-translational histone modifications—including methylation (e.g., H3K4me3, H3K9me3) and acetylation (e.g., H3K9ac, H3K27ac)—alter the degree of chromatin condensation and thereby influence gene expression without changing the DNA sequence itself. These modifications are dynamic and are crucial for normal placental function and the development of trophoblast cells mediating the interaction between mother and fetus during pregnancy [128].

Changes in histone marks are associated with placental function as they affect the expression of growth factors, trophoblast cell invasiveness, and vascularization—key processes that allow the transfer of nutrients and oxygen from the mother to the fetus. For instance, H3K4me3 and H3K9ac are generally associated with active gene transcription, while increased methylation of certain marks can be associated with repressed chromatin, which is demonstrated to be deregulated in complicated pregnancies such as preeclampsia [129].

Histone epigenetic regulation does not function in isolation but through a coordinated interaction of enzymes that add modification groups (“writers”), enzymes that remove these modifications (“erasers”), and factors that recognize them (“readers”). This complex allows chromatin remodeling in response to nutritional, oxidative, and hormonal signals during pregnancy, supporting proliferation and differentiation of placental and fetal cell lineages [130].

Epigenetic patterns are also transmitted to the fetus: histone modification patterns influence the expression of genes involved in proliferation, differentiation, and tissue organization in organs such as the brain, liver, and immune system. Disruption of these patterns, caused by intrauterine stress or oxidative factors, is associated with adverse perinatal outcomes, including preterm birth, growth restriction, and long-term metabolic dysfunctions [131].

Telomeres—repetitive nucleotide sequences at chromosome ends—are crucial for maintaining genomic integrity and regulating cellular senescence. During intensive fetal cell division, telomere length remains relatively long but progressively shortens with each division and under oxidative stress. This process is also observed in the placenta, where telomere shortening signals replicative tissue senescence and participates in gestational adaptations that prepare the organism for labor and other dynamic changes during pregnancy [132]. Telomere status is directly associated with perinatal outcomes, as telomere shortening in the placenta and umbilical cord blood correlates with intrauterine growth restriction, spontaneous abortion, and various pregnancy complications. Mechanisms driving telomere shortening induce cellular aging and reactive changes that can compromise placental function and fetal development, while high oxidative stress further accelerates these processes [133].

**Figure 2:**
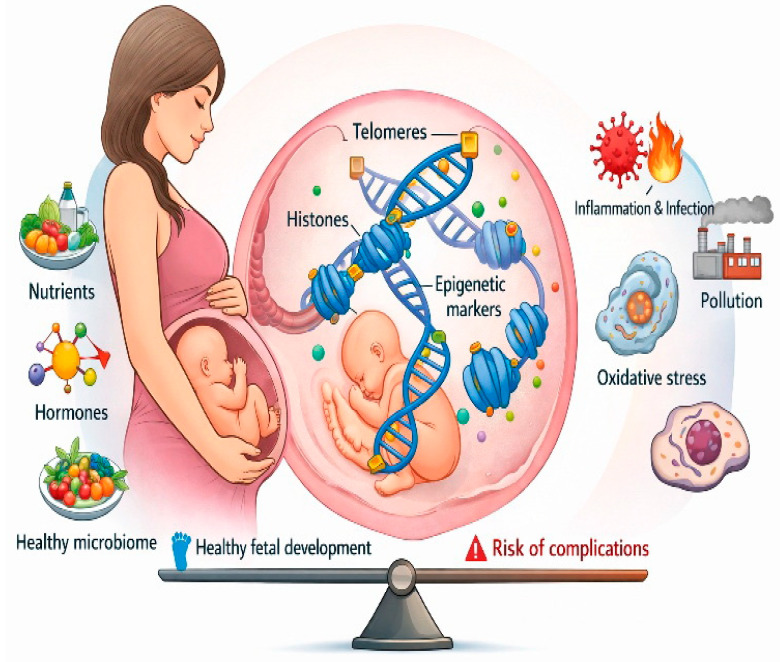
Maternal–Fetal Epigenetic Interactions Shaping Perinatal Outcomes.

Maternal factors, including nutritional conditions, oxidative and inflammatory status, and physiological stress during pregnancy, influence fetal telomere dynamics. Studies indicate that prenatal exposure to stress factors and microbiome imbalance can result in shorter telomeres in neonates, which is associated with developmental and cardiometabolic risks later in life, within the framework of fetal programming [134]. Furthermore, different placental components, such as trophoblast subtypes, show specific patterns of epigenetic modifications that dynamically change throughout gestation. These changes are also reflected in global patterns where certain histone marks decrease or increase as pregnancy progresses, and abnormal values are associated with complications such as preeclampsia and growth restriction [135]. Epigenetic mechanisms involving histone modifications and telomere dynamics represent an integrated network that allows the placenta and fetus to respond to nutrients, oxidative conditions, and hormonal signals. Disturbances in these mechanisms—through microbiome dysbiosis, oxidative stress, nutritional deficits, or prenatal challenges—are associated with adverse perinatal outcomes and potentially with lasting changes in offspring health, in line with the concept of fetal programming [136]. It can be said that modifications and telomere dynamics serve as epigenetic mediators between the maternal external environment and the fetus’s internal developmental programs, enabling gene regulation and cellular processes essential for normal growth, adaptation, and perinatal outcome [137] (Figure 2).

Maternal environment influences fetal development through epigenetic mechanisms, histones, telomeres, and epigenetic markers representing regulation of gene expression without changes in DNA sequence. Beneficial maternal factors—nutrients, hormones, and a healthy microbiome—support optimal fetal development, while adverse exposures such as inflammation, infection, oxidative stress, and pollution disrupt epigenetic programming and increase the risk of complications. The balance scale illustrates the dynamic equilibrium between protective and harmful influences shaping perinatal outcomes.

## 5. Nutritional Factors and Their Molecular Interactions

Maternal nutrition constitutes a cornerstone determinant of perinatal outcomes, shaping fetal growth, immune system development, and metabolic programming through complex molecular, epigenetic, and microbiome-mediated pathways. The integrated effects of macronutrients (proteins, lipids, carbohydrates, dietary fibers), vitamins, trace elements, bioactive compounds, omega-3 polyunsaturated fatty acids (PUFAs), probiotics, and prebiotics orchestrate the intrauterine environment, modulating oxidative stress, lipid metabolism, immune tolerance, and epigenetic regulation [138]. Nutrients act both directly, by influencing intracellular signaling pathways, transcription factors, and enzyme activity, and indirectly, via modulation of the maternal and fetal microbiome [139]. Understanding these pathways provides a mechanistic foundation for optimizing perinatal outcomes and long-term health trajectories in offspring [140].

### 5.1. Proteins and Amino Acids

Proteins and their constituent amino acids are essential for fetal growth and developmental programming. Amino acids are not only building blocks for proteins but also participate in biosynthetic pathways, cell proliferation, and metabolic signaling that support placental and fetal development [141].

Arginine, a member of the arginine family of amino acids, plays a particularly important role in promoting fetal growth; prenatal supplementation with arginine or related compounds has been associated with improved birth outcomes, such as increased birth weight ratios in complicated pregnancies, reflecting enhanced nutrient supply and placental function [142]. Arginine serves as a precursor for nitric oxide (NO) synthesis via nitric oxide synthase, and NO influences vascular function and uteroplacental blood flow, which are critical determinants of nutrient delivery to the fetus [143].

Plasma concentrations of specific amino acids during pregnancy, including glutamine, have been linked to neonatal anthropometric outcomes, where higher maternal glutamine levels early in gestation correlate with increased birthweight indices, suggesting that amino acid availability may serve as a biomarker of fetal growth and metabolic status. Essential amino acids and their transport across the placenta contribute to fetal protein accretion and energy balance, while alterations in maternal amino acid metabolism are observed in conditions such as gestational diabetes mellitus, indicating disrupted nutrient handling that may impact both maternal and fetal metabolic pathways [144].

Beyond direct metabolic roles, nutrients involved in one-carbon metabolism (such as methionine, folate, and related methyl donors) provide methyl groups necessary for DNA methylation and other epigenetic modifications, linking maternal nutrition to epigenetic regulation during development [145]. Although most research focuses on folate and choline, one-carbon metabolites, including methionine, influence the pool of S-adenosylmethionine (SAM), the universal methyl donor for DNA and histone methylation, suggesting a mechanism by which maternal nutrient status can modulate gene expression programs relevant to fetal growth and long-term health trajectories [146].

### 5.2. Lipids and Fatty Acids

Lipids are essential for fetal development, contributing to membrane structure, cellular signaling, and energy metabolism during gestation [147]. Maternal intake of long-chain omega-3 polyunsaturated fatty acids (EPA and DHA) modulates maternal–fetal lipid dynamics and inflammatory responses. Supplementation with omega-3 PUFAs has been associated with increased gestational length, reduced risk of preterm birth, and higher birth weight [148].

Clinical trials indicate that omega-3 PUFA intake reduces placental and maternal inflammatory markers. For instance, overweight and obese pregnant women receiving EPA and DHA showed decreased expression of TLR4, IL-6, IL-8, and TNF-α in placental tissue compared with controls [149]. Placental LC-PUFA levels, particularly DHA, increase with supplementation, correlating with maternal and cord blood DHA concentrations and elevated precursors of specialized pro-resolving lipid mediators [150]. Maternal DHA supplementation also modifies placental nutrient transporter expression, including increased fatty acid transport protein 4, while reducing placental inflammation and amino acid transporter activity, particularly in pregnancies complicated by obesity [151]. These findings indicate that maternal omega-3 status influences placental nutrient handling and inflammatory profiles, which may impact fetal growth and neurodevelopmental pathways. Epigenetic analyses reveal that maternal LC-PUFA status is associated with placental DNA methylation and histone acetylation patterns, highlighting the potential for modulation of gene expression relevant to angiogenesis and fetal development [152].

In contrast, excessive maternal intake of saturated fats is associated with proinflammatory placental environments. High circulating palmitic acid levels correlate with increased NF-κB-mediated expression of IL-6 and TNF-α, mitochondrial depolarization, and caspase-3 activation in placental trophoblasts. Monounsaturated fatty acids mitigate these effects by inhibiting NF-κB signaling and reducing lipoapoptosis [153]. Overall, maternal fatty acid composition—adequate omega-3 intake combined with balanced saturated and monounsaturated fats—is crucial for placental function, inflammation regulation, and optimal fetal development [154].

### 5.3. Carbohydrates and Dietary Fibers

Carbohydrate quality and quantity significantly impact maternal glucose homeostasis, fetal insulin signaling, and epigenetic programming. Complex carbohydrates with low glycemic index stabilize maternal glucose levels, reducing hyperglycemia-induced oxidative stress and NF-κB activation in fetal tissues [155]. Dietary fibers, including soluble fibers such as inulin and resistant starch, are fermented by gut microbiota to produce SCFAs (acetate, propionate, butyrate), which inhibit HDACs, enhancing chromatin accessibility and transcription of anti-inflammatory and antioxidant genes. SCFAs also activate Nrf2, upregulating antioxidant response elements (AREs) and phase I/II detoxifying enzymes [156]. Reduced fiber intake diminishes SCFA production, increasing susceptibility to oxidative stress, dysregulated lipid metabolism, and immune imbalance [157].

### 5.4. Vitamins and Micronutrients

Vitamins and trace elements serve as cofactors for enzymes regulating epigenetic modifications, redox homeostasis, and immune signaling [32]. Vitamin D, through the vitamin D receptor (VDR) and retinoid X receptor (RXR) complex, binds vitamin D response elements (VDREs) to upregulate anti-inflammatory cytokines IL-10, TGF-β) and suppress NF-κB and TNF-α signaling, reducing oxidative stress [49,158,159]. Folate and vitamin B12 act as methyl donors via SAM, supporting DNA and histone methylation in genes involved in lipid metabolism, antioxidant defense (SOD, GPx, CAT), and immune tolerance [160]. Selenium, as a cofactor for GPx and other selenoproteins, stabilizes Nrf2 and regulates epigenetic patterns, protecting against ROS-mediated damage [161,162]. Zinc and iron act as cofactors for DNA methyltransferases (DNMTs) and histone methyltransferases (HMTs), modulating immune and metabolic gene expression [163,164].

### 5.5. Polyphenols and Bioactive Compounds

Dietary polyphenols, including flavonoids and phenolic acids, play a key role in maintaining cellular redox balance by acting as powerful antioxidants and regulators of oxidative stress. They boost the cell’s ability to neutralize ROS, thus shielding against oxidative damage [165]. A main way polyphenols achieve this is by activating Nrf2. Once activated, Nrf2 binds to the antioxidant response element (ARE) in target gene promoters, promoting the transcription of phase II detoxifying and antioxidant enzymes. This regulation is further refined through epigenetic changes, such as DNA methylation and histone modifications at the Nrf2 promoter, enabling polyphenols to sustain an antioxidant response [166]. Beyond their function in redox control, polyphenols actively influence inflammatory signaling pathways. By inhibiting nuclear factor-κB (NF-κB) and (MAPK) cascades, they decrease the production of pro-inflammatory cytokines, thereby reducing chronic inflammatory processes [167]. At the same time, these compounds affect the epigenetic landscape by modulating DNA methyltransferases (DNMTs), HDACs, and histone acetyltransferases (HATs), which collectively shape chromatin structure and transcriptional programs that control metabolic, immune, and antioxidant pathways [168]. Certain polyphenols, including curcumin, resveratrol, and epigallocatechin-3-gallate (EGCG), have been shown to directly interact with these epigenetic enzymes, emphasizing their ability to connect dietary exposure with gene regulatory networks [169].

The interplay between polyphenols and the gut microbiota further amplifies their systemic effects. Microbial metabolism transforms polyphenols into SCFAs. such as butyrate, propionate, and acetate, which in turn act as epigenetic regulators, influencing DNA methylation, histone modifications, and non-coding RNA expression, thereby modulating host gene expression and regulating immune and metabolic functions [170]. Beyond their epigenetic roles, SCFAs engage G-protein-coupled receptors (GPCRs), integrating microbial-derived signals with host metabolic and immune responses [171].

Taken together, these molecular and microbiota-mediated pathways converge to provide a coherent framework by which dietary polyphenols exert pleiotropic biochemical and immunometabolic effects. By simultaneously modulating oxidative stress, inflammation, epigenetic regulation, and host–microbiome interactions, polyphenols represent a key dietary interface for maintaining physiological resilience and health [45].

### 5.6. Probiotics and Prebiotics

The maternal gut microbiome plays a significant role in the fermentation of plant fibers and the production of metabolites such as SCFAs, acetate, propionate, and butyrate. These metabolites act as signaling molecules that modulate maternal metabolic processes and immune function during pregnancy. SCFAs influence energy homeostasis, lipid metabolism, and inflammatory pathways, and are increasingly recognized for their potential to indirectly influence fetal development by altering maternal physiology [172].

Clinical cohort studies have shown that women with metabolic complications of pregnancy, including gestational diabetes mellitus (GDM) and preeclampsia, often display altered SCFA profiles in their circulation. Specific SCFA concentrations in these pregnancies differ significantly from those in healthy controls and are associated with changes in placental metabolic activity and maternal inflammatory markers, indicating a link between maternal microbial metabolites and adverse pregnancy outcomes [173].

Experimental studies suggest that prebiotic intake during pregnancy can modify the maternal microbiome, enhancing SCFA production and influencing immune cell populations in gestational tissues. Such effects point toward a mechanism through which maternal microbiota may contribute to fetal immune tolerance and early-life immune programming [32]. Moreover, systematic reviews indicate that maternal consumption of probiotics, prebiotics, or synbiotics can positively shape neonatal gut colonization, particularly increasing Bifidobacterium abundance in infants delivered by cesarean section, although optimal protocols remain under investigation [49].

**Figure 3:**
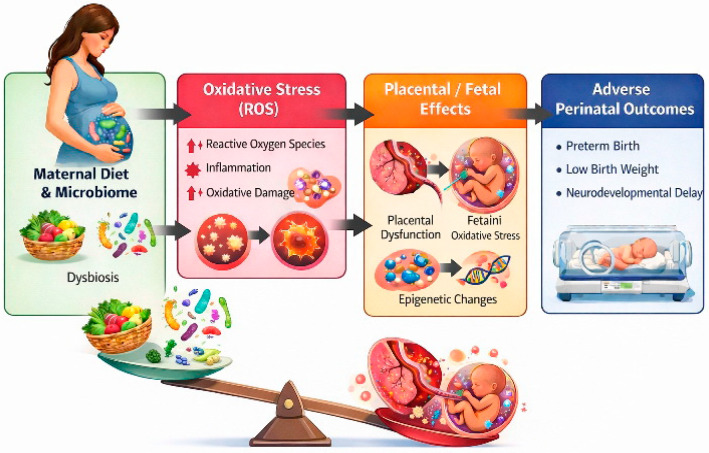
The Role of Maternal Nutrition in Shaping Perinatal Outcomes.

Finally, epidemiological data suggest that disruptions in maternal SCFA production and gut microbiota composition correlate with an increased risk of pregnancy complications, such as preeclampsia. These findings underscore the importance of understanding maternal–microbiota metabolic interactions to improve maternal and fetal health outcomes [174] (Figure 3).

Imbalanced maternal nutrition significantly modulates the composition and functional dynamics of the gut microbiome, contributing to dysbiosis and inducing oxidative stress with increased production of ROS. ROS cause oxidative damage to membranes, proteins, and lipids in the placenta and fetus, simultaneously activating inflammatory processes that modulate intrauterine development. These molecular and cellular alterations may impact perinatal outcomes, including preterm birth, reduced birth weight, and potential changes in fetal neurodevelopment, reflecting the complex interplay between nutritional, microbiome, and oxidative–inflammatory factors during pregnancy.

## 6. Conclusions

Maternal diet and gut microbiome composition critically determine intrauterine conditions, modulating fetal development through metabolic, immune, and epigenetic pathways. Nutrients and microbiome-derived metabolites regulate placental and fetal gene expression, immune signaling, and oxidative balance, whereas dysbiosis or suboptimal nutrition increases inflammation and oxidative stress, impairing angiogenesis, nutrient transport, and fetal growth. These integrated mechanisms highlight the central role of maternal environmental quality in shaping perinatal outcomes and long-term neonatal health, emphasizing the potential of targeted nutritional and microbiome-based interventions to optimize fetal and postnatal development.

## Data Availability

No new data were created or analyzed in this study. Data sharing is not applicable to this article.
